# Syphilis: Its Diagnosis and Treatment

**Published:** 1901-12

**Authors:** 


Syphilis: Its Diagnosis and Treatment. By William S. Gottheil, M.D., Professor of Dermatology and Syphilology, New York School of Clinical Medicine; Dermatologist to the Lebanon and Beth-Israel Hospitals, the West-Side German Dispensary, etc. Profusely illustrated. Pages 216. Chicago: G. P. Engelhard & Company. 1901. [Price, $1.00 net.]

Dr. Gottheil has brought out a book of 216 pages carrying the above title. The work deals only with well-recognised facts concerning the evolution, course and treatment of the disease, beginning with a historical introduction. There is so little said in each chapter about the particular feature discussed that the general practitioner may, at times, find himself bewildered instead of assisted. The portion specially devoted to treatment is likely to serve him best. It is fair to say, however, that it would be utterly impossible to treat so fruitful a subject in the limited space here devoted to it. The photographic reproductions, which number twenty-eight, compose the most valuable feature of the work, being unusually strong and striking. The utility of such pictures cannot be overestimated.

The presswork and binding have been well executed: however, from the general appearance of the work, it may easily be mistaken for a modern novelette. G. W. W.

